# Diameter of the fetal pancreas and abdomen-to-pancreas-ratio: novel ultrasound parameters in fetal growth restriction

**DOI:** 10.1007/s00404-026-08339-w

**Published:** 2026-02-16

**Authors:** Lotta von der Gathen, Janina Braun, Mareike Möllers, Chiara De Santis, Daniela Willy, Rene Schmidt, Kathleen Oberste, Ralf Schmitz, Kathrin Oelmeier

**Affiliations:** 1 Department of Gynecology and Obstetrics, University Hospital Münster, Albert-Schweitzer-Campus 1, 48149 Munster, Germany; 2 Institute of Biostatistics and Clinical Research, University of Münster, Munster, Germany; 3 Department of Gynecology and Obstetrics, Florence-Nightingale-Hospital, Düsseldorf, Germany

**Keywords:** Fetal growth restriction, Pancreatic diameter, Prenatal ultrasound, Fetal organ mapping

## Abstract

**Purpose:**

The aim of this study was to compare fetal pancreas size at second trimester ultrasound screening of growth-restricted and normal weight fetuses.

**Methods:**

One hundred sixty-six fetuses between 18 + 0 and 21 + 6 weeks of gestation were included in this retrospective study. 83 fetuses with a birth weight below the 10th centile were included in the study group which was further subdivided into two subgroups depending on the presence (subgroup 1) or absence (subgroup 2) of prenatal signs of fetal growth restriction. The control group consisted of 83 normal fetuses matched for sex and gestational age at examination. The pancreatic diameter (PD) was measured in a standard 2D plane of the fetal abdomen. Statistical analyses comprised descriptive statistics, reliability testing, and multivariable modelling to explore group differences and covariate effects on pancreatic diameter.

**Results:**

The diameter of the pancreas was increased in the LBW group compared to the control group [3.7 mm vs. 3.1 mm (*p* < 0.001)]. The ratio of abdominal circumference (AC) to pancreatic diameter was significantly smaller in the LBW group [41.51 vs. 50.62 (*p* < 0.001)]. The result was consistent in the subgroup analysis. The difference of the median PD and ratio of AC/PD is greatest in subgroup 1 compared to the control group [PD: 4.2 mm vs. 3.1 mm (*p* < 0.001) and AC/PD-ratio 35.33 vs. 51.88 (*p* < 0.001)].

**Conclusion:**

The diameter of the fetal pancreas, as measured in this study, is a valuable parameter for the detection of small for gestational age and growth-restricted fetuses. Further studies are needed to further validate our results and their implication for clinical decision-making.

## What does this study add to the clinical work?

**Table:** 

Detection of fetal growth restriction (FGR) and distinction between FGR and small for gestational age (SGA) fetuses is one oft he major challenges in obstetrics today. Prenatal ultrasound of fetal organs such as the size of the fetal pancreas and its relation to the abdominal circumference can be helpful tools in this situation.	

## Introduction

Fetal growth restriction (FGR) is one of the major challenges in prenatal medicine today. It affects up to 10% of all pregnancies [1] and is associated with increased perinatal morbidity and mortality [2, 3], as well as an increased risk of chronic disease later in life [4].

FGR is defined as the failure of the fetus to reach its genetic growth potential [1]. The most frequent cause of FGR is chronic placental insufficiency [5]. FGR is divided into early-onset FGR and late-onset FGR, with the demarcation at 32 weeks’ gestation [6]. In clinical practice, it is often defined by an estimated fetal weight (EFW) below the 3rd, 5th or 10th centiles even though some fetuses growing below the 10th centile can be considered constitutionally small for gestational age (SGA) without an increased risk for poor perinatal outcome [7, 8]. While first and second trimester screenings can detect up to 90% of the early-onset FGR using uterine Doppler velocimetry, fetal biometric parameters and maternal characteristics, the distinction between late-onset FGR and SGA fetuses remains challenging [9].

In addition to biometric parameters, so-called organ mapping has become increasingly important in recent years. Various organs, such as the fetal heart, thymus or adrenal gland have been shown to differ in size or morphology in prenatal ultrasound as indicators for fetal complications, malformations or genetic disease [10–12]. In FGR, chronic restriction of fetal oxygen and nutrient supply has been shown to affect fetal circulation, the physiology of developing organs as well as the fetal metabolism ([4, 13]).

The fetal pancreas is a composite organ derived from a dorsal and a ventral bud of the distal foregut endoderm. Its development begins on day 29 of gestation and the two buds fuse during gut rotation at 6–7 weeks of gestation [14]. Animal studies have shown that the fetal pancreas initiates its endocrinological activity shortly after the fusion of the two buds, and therefore influences the fetal development and glucose regulation [15, 16].

Sonographic visualisation of the fetal pancreas was first described in 1988 [17]. Since then, a small number of studies have investigated the size of the fetal pancreas [18, 19]. An increased size of the pancreas was found in fetuses exposed to maternal gestational diabetes [20–22]. Another study found that increased fetal pancreas size was associated with composite adverse neonatal outcomes in singleton pregnancies including normal and FGR fetuses [23].

In this study, we aimed to assess whether the size of the fetal pancreas differs in SGA and FGR fetuses in comparison to normal fetuses as a possible diagnostic marker for the early detection of SGA or FGR fetuses.

## Methods

### Study design

This retrospective, single-center study was conducted at the Department of Gynecology and Obstetrics, University Hospital Münster, Germany between 2012 and 2023. It was approved by the Ethics Committee of the local medical council (2023-290-f-S) and designed in accordance with the Declaration of Helsinki.

All ultrasound examinations were performed by specialists in prenatal diagnostics using high-end ultrasound machines (Philips iU22 and Epiq Systems with C9-1 and C5-1 transducers). The fetal pancreas was visualized in the axial view of the upper abdomen between 18 + 0 and 21 + 6 weeks of gestation. Gestational age was calculated from the crown-rump length at the first trimester screening of each fetus.

We included singleton pregnancies with complete follow-up and satisfactory image quality of the fetal pancreas at second trimester scan. We excluded multiple pregnancies and fetuses with a diagnosis or the suspicion of congenital fetal anomalies, aneuploidy or sonographic evidence of malformations.

Fetuses with a birth weight below the 10th percentile composed the study group, now referred to as low birthweight (LBW) group. Each fetus in the control group was matched for sex and day of examination to minimize the influence of sex and fetal age on the size of the pancreas.

The LBW group was categorized in two subgroups for subgroup analysis. Subgroup 1 included all fetuses, that showed prenatal signs of FGR at time of examination. Signs of FGR were defined by an estimated fetal weight below the third percentile and/or a uterine artery pulsatile index above the 95th percentile suggesting an increased utero-placental resistance. Subgroup 2 consisted of all the remaining LBW fetuses without the signs of FGR at time of examination.

**Fig. 1:**
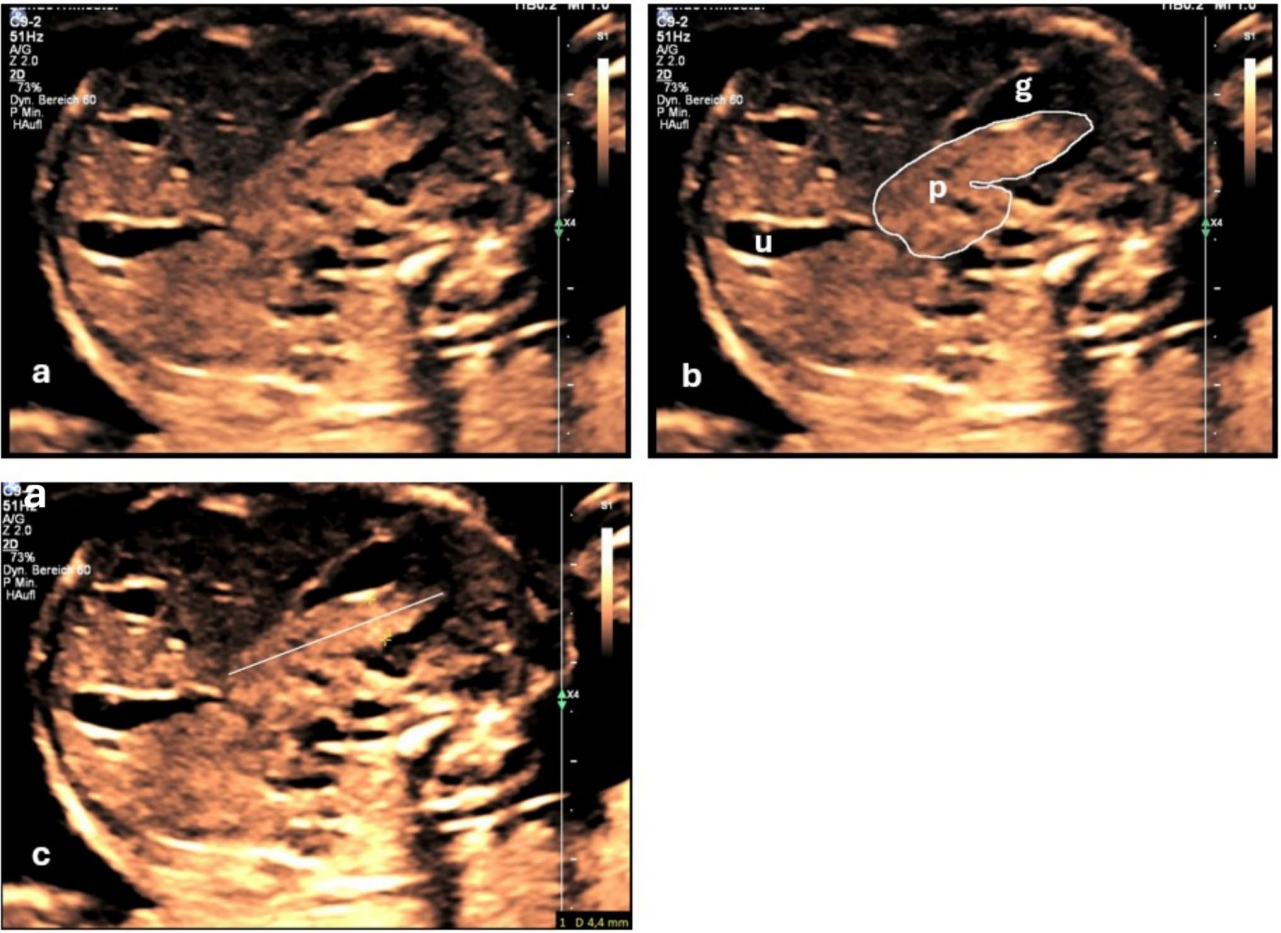
Abdominal cross-section at 20 + 6 weeks gestation, overview (**a**)**,** outline of the pancreas **(b**) and pancreas measurement (**c**): the part of the pancreas adjacent to the gastric cavity, diameter perpendicular to the long axis of the pancreas (blue dotted line) with calipers set upon the inner edge of the pancreatic parenchyma (yellow calipers). g, gastric cavity; p, pancreas; u, umbilical vein

The fetal pancreas was measured as depicted in Fig. 1: First, the sigmoidal part of the pancreas was visualized in the abdominal cross-section at the level of the gastric cavity. Once a satisfactory visualization was achieved, the part of the pancreas adjacent to the gastric cavity was measured. To this end, we measured the diameter of the pancreas perpendicular to the long axis of the pancreas with calipers set to touch the inner edge of the pancreatic parenchyma.

Clinical parameters were obtained from the hospital database, including abdominal circumference (AC), head circumference (HC), estimated fetal weight (EFW), uterine artery pulsatile index left and right at the time of examination, pregnancy outcome with APGAR score at 5 min, birth weight, umbilical cord pH, and possible maternal confounders such as maternal body mass index (BMI), maternal smoking and maternal chronic disease, more precise hypertension and/or diabetes.

### Statistical analysis

Descriptive statistics were used to describe the study population. Metric parameters were described using median and interquartile range (IQR; 25–75% quantile), while categorical parameters were expressed as frequencies.

To compare a metric or binary parameter between the LBW group and the control group, we used the Wilcoxon signed-rank test or McNemar’s test, respectively, to account for matching. We generated box plots and a scatter plot to visualize group differences in the distribution of pancreatic diameters and the association between pancreas diameter and gestational age.

Intraclass correlation coefficients (ICC) were used to quantify the interrater and intrarater reliability of diameter measurements [24]. In a randomly selected subgroup of 20 cases, two observers measured each of the pancreatic diameters, one of them at two different times. Interrater reliability of diameter measurements was quantified by intraclass correlation coefficient ICC (3,1). Intrarater reliability of diameter measurements was quantified by intraclass correlation coefficient ICC (1,1).

A multivariable linear mixed model was performed to examine the influence of different covariates on the pancreatic diameter. The following covariates were included in the analysis: low birth weight (binary: yes/no, reference: no), maternal BMI (metric, kg/m^2^), maternal smoking (binary: yes/no, reference: yes), and maternal chronic diseases (binary: yes/no, reference: yes). A two-by-two unstructured covariance structure was used to account for matching the LBW and control groups.

Due to the retrospective nature of the study, all statistical analyses were exploratory and thus hypothesis-generating only. *p*-values are considered as descriptive measures to indicate potentially relevant effects, with a threshold of *p* < 0.05 for statistically significant effects. For the statistical analysis we used IBM SPSS Statistics (Version 29.0.2.0, IBM Corporation, Armonk, NY, USA).

## Results

We included 166 fetuses in our study, with 83 in the LBW group and 83 in the control group. 25 of the LBW fetuses showed signs of a FGR at the time of the ultrasound screening, with an EFW below the third percentile and/or an increased utero-placental resistance, and were included in subgroup 1. The other 58 fetuses without signs of FGR formed subgroup 2.

The characteristics of the LBW fetuses and the control groups are shown in Table 1. The median gestational age at examination was 20.48 weeks. The two groups did not differ in APGAR score at 5 min, umbilical artery PH, maternal BMI and presence of maternal diabetes. Gestational age at birth was noticeably lower in the LBW group. However, in terms of absolute numbers, gestational age at birth was above 37 weeks of gestation in both groups.

**Table 1:** Clinical characteristics and metric data at the time of ultrasound screening of the low birth weight (LBW) group and control group

Parameter (ultrasound screening)	LBW group (n = 83)	Control Group (n = 83)	*p* value
GA at screening, weeks	20.5 (1.0)	20.5 (1.0)	1.000
Pancreas diameter (PD), mm	3.7 (1.0)	3.1 (0.8)	< 0.001
Abdominal circumference (AC), mm	149.3 (17.4)	155.7 (17.9)	< 0.001
Head circumference (HC), mm	172.8 (16.1)	177.9 (14.8)	< 0.001
Estimated fetal weight (EFW), g	341.0 (78.0)	371.0 (90.0)	< 0.001
Pulsatile index (PI) uterine a.(left)	1.01 (0.64)	0.87 (0.39)	0.013
Pulsatile index (PI) uterine a. (right)	1.08 (0.64)	0.91 (0.3)	0.007
Ratio AC/PD	41.51 (10.8)	50.62 (11.79)	< 0.001
*Parameter (outcome)*			
GA at birth, weeks	37.6 (1.7)	39.2 (0.9)	< 0.001
5-min APGAR	10 (1.0)	10 (1.0)	0.067
pH umbilical artery	7.3 (1.0)	7.29 (1.2)	0.246
Birth weight, g	2570 (640)	3555 (255)	< 0.001
*Parameter (maternal confounders)*			
Maternal BMI	24.45 (6.21)	25.2 (6.23)	0.188
Maternal diabetes	3 (3.6%)	2 (2.4%)	0.655
Maternal hypertension	9 (10.8%)	3 (3.6%)	0.146
Maternal smoking	9 (10.8%)	1 (1.2%)	0.021

Data presented as median (IQR: 25–75% quantile), *p* values of Wilcoxon signed-rank test for metric outcome and McNemar’s test for binary outcome

Abdominal circumference (AC), head circumference (HC) and estimated fetal weight (EFW) were smaller in the LBW group than in the control group (149.3 [17.4] vs. 155.7 [17.9], *p* < 0.001); (172.8 [16.1] vs. 177.9 [14.8], *p* < 0.001); and (341.0 [78.0] vs. 371.0 [90.0], *p* < 0.001), respectively. In addition, left and right uterine artery pulsatile index and the incidence of maternal smoking were higher in the LBW group than in the control group (1.01 [0.64] vs. 0.87 [0.39], *p* = 0.013); (1.08 [0.64] vs. 0.91 [30.0], *p* = 0.007); (9 [10.8%] vs. 1 [1.2%], *p* = 0.021).

**Fig. 2:**
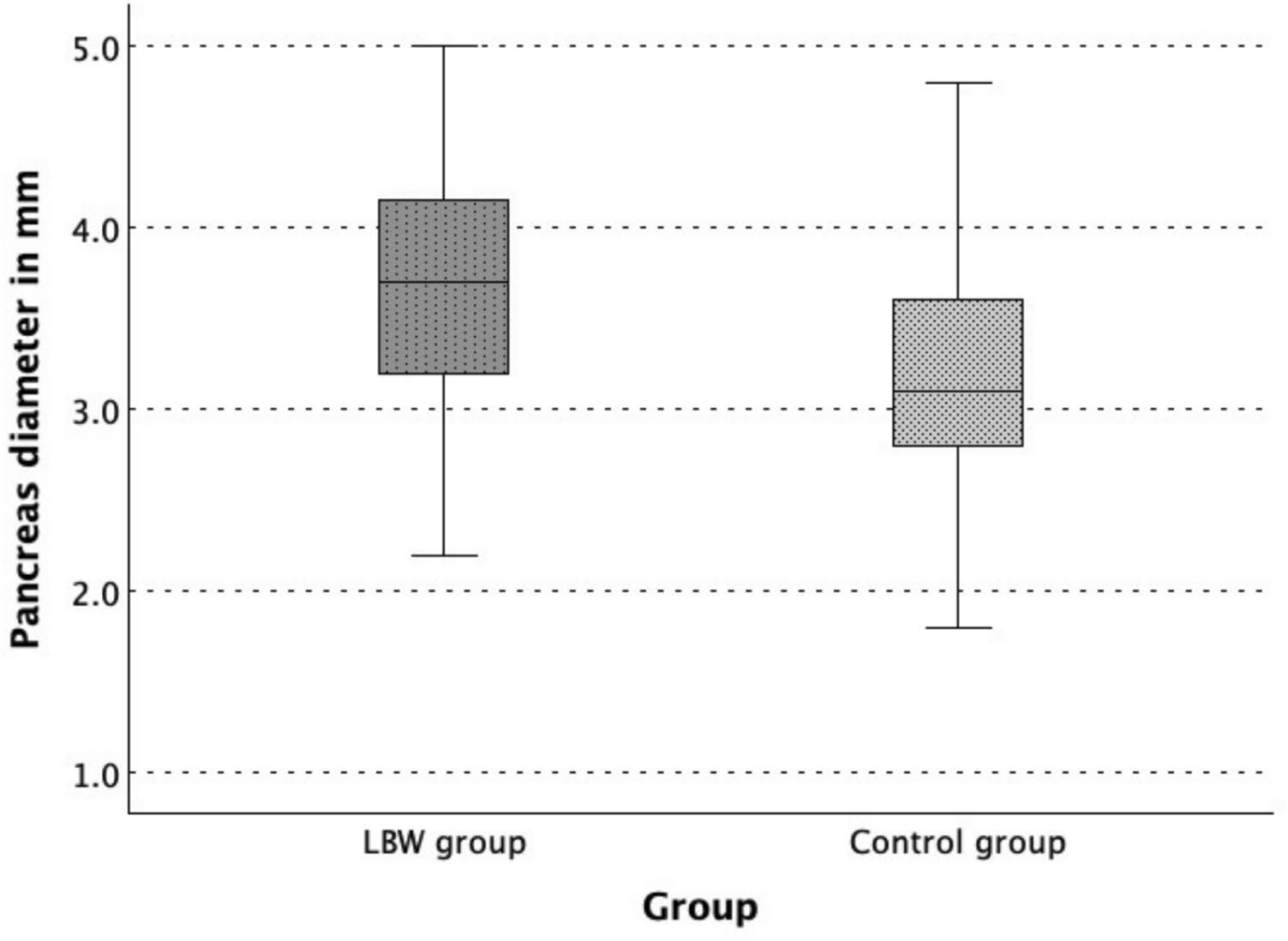
Boxplot of the pancreas diameter in the low birth weight (LBW) group (n = 83) and control group (n = 83)

The pancreas diameter (PD) was increased in the overall LBW group (3.7 [1.0]) compared to the control group (3.1 [0.8], *p* < 0.001) and consequently, the ratio of AC to PD was lower (41.51 [10.8] vs. 50.62 [11.79], *p* < 0.001). Figure 2 illustrates the comparison of the pancreas diameter in the LBW group and the control group.

**Fig. 3:**
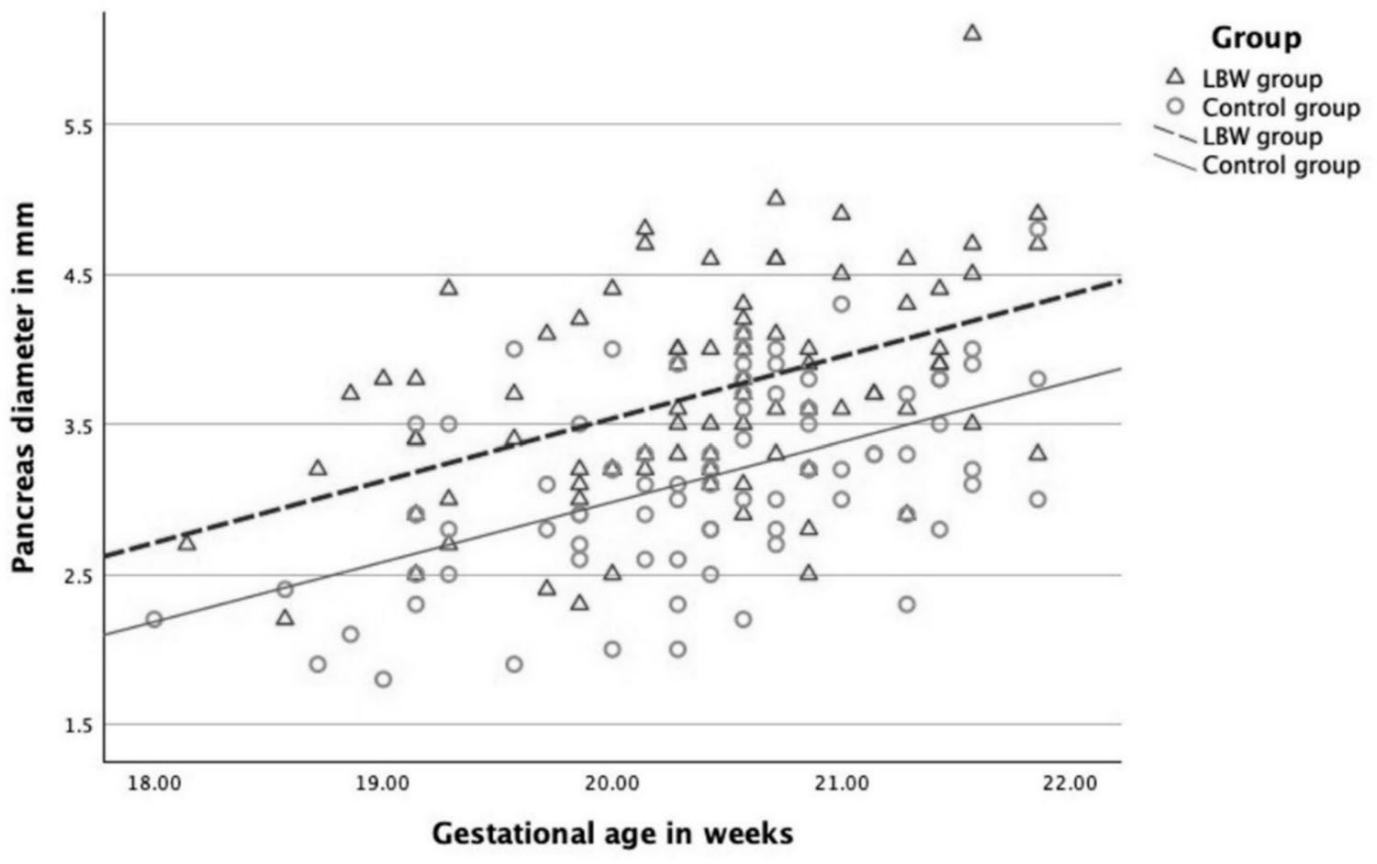
Grouped scatterplot of the pancreas diameter and gestational age at screening in the low birth weight (LBW) group (n = 83) and control group (n = 83)

The correlation between pancreas diameter and gestational age at screening is presented in Fig. 3.

**Fig. 4:**
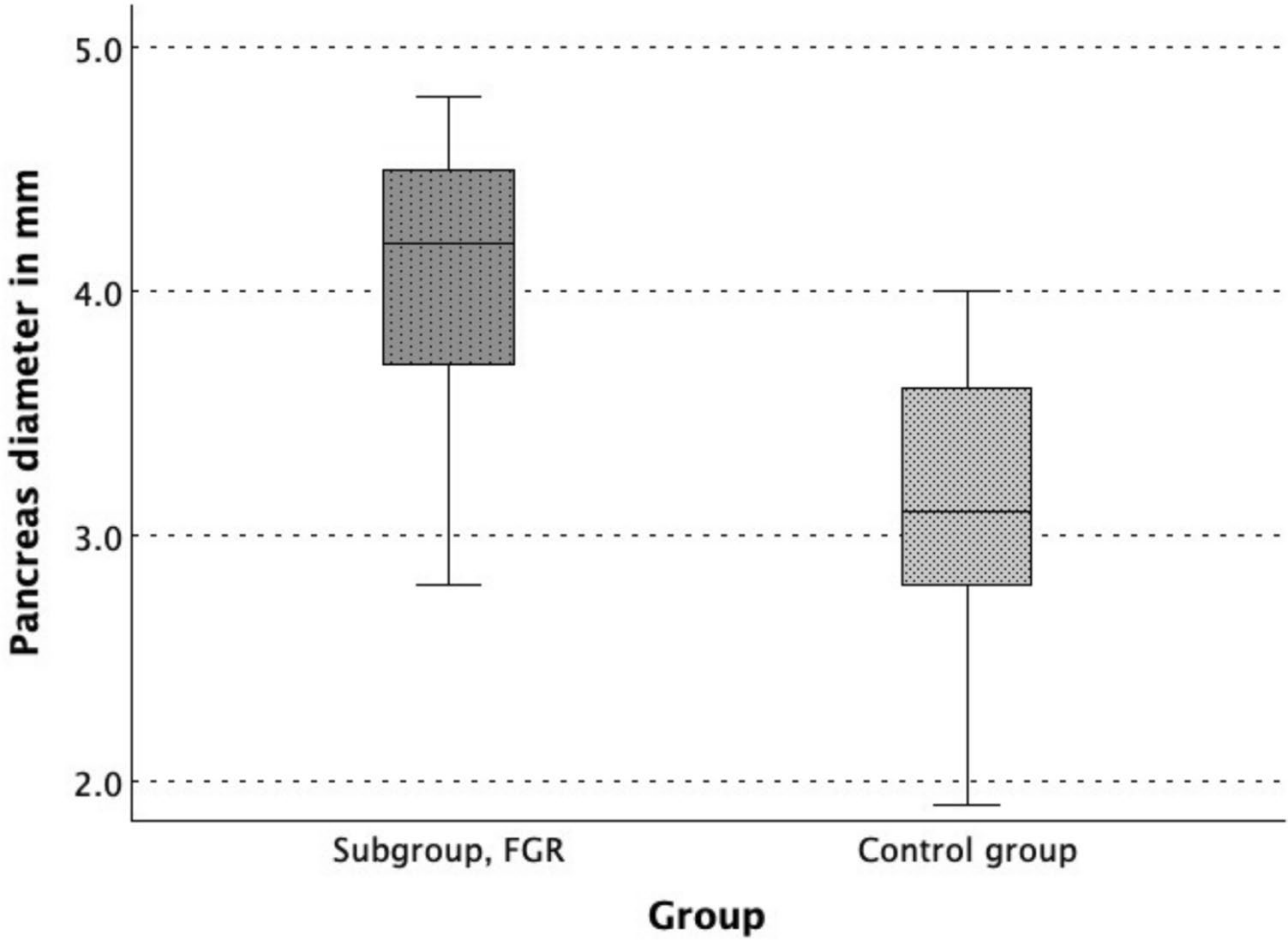
Boxplot of the pancreas diameter of subgroup 1, with fetal growth restriction (FGR) (n = 25), in comparison to the corresponding matched control subgroup (n = 25)

These results were consistent in subgroup analysis (Table 2 and 3). As shown in Fig. 4, pancreas diameter was increased in subgroup 1 (4.2 [0.8]) compared to the control group (3.1 [1], *p* < 0.001). In subgroup 2, without prenatal signs of FGR, the pancreas diameter was also increased (3.5 [0.9] vs. 3.2 [0.8], p 0.003).

**Table 2:** Metric data at the time of ultrasound screening of subgroup 1, with fetal growth restriction (FGR), and the control group

Parameter (ultrasound screening)	Subgroup 1, FGR (n = 25)	Control group (n = 25)	*p* value
Pancreas diameter (PD), mm	4.2 (0.8)	3.1 (1.0)	< 0.001
Abdominal circumference (AC), mm	149.2 (14.4)	155.4 (12.6)	0.002
Estimated fetal weight (EFW), g	344.0 (62.5)	382.0 (81.0)	0.004
Ratio AC/PD	35.33 (7.31)	51.88 (11.39)	< 0.001

Data presented as median (IQR: 25–75% quantile), *p* values of Wilcoxon signed-rank test

**Table 3:** Metric data at the time of ultrasound screening of subgroup 2, without signs of fetal growth restriction (FGR), and the control group

Parameter (ultrasound screening)	Subgroup 2, no signs of FGR (n = 58)	Control group (n = 58)	*p* value
Pancreas diameter (PD), mm	3.5 (0.9)	3.2 (0.8)	0.003
Abdominal circumference (AC), mm	150.0 (23.0)	156.3 (19.4)	0.001
Estimated fetal weight (EFW), g	340.0 (83.0)	364.0 (91.3)	0.001
Ratio AC/PD	43.42 (10.71)	50.25 (12.29)	< 0.001

Data presented as median (IQR: 25–75% quantile), *p* values of Wilcoxon signed-rank test

**Fig. 5:**
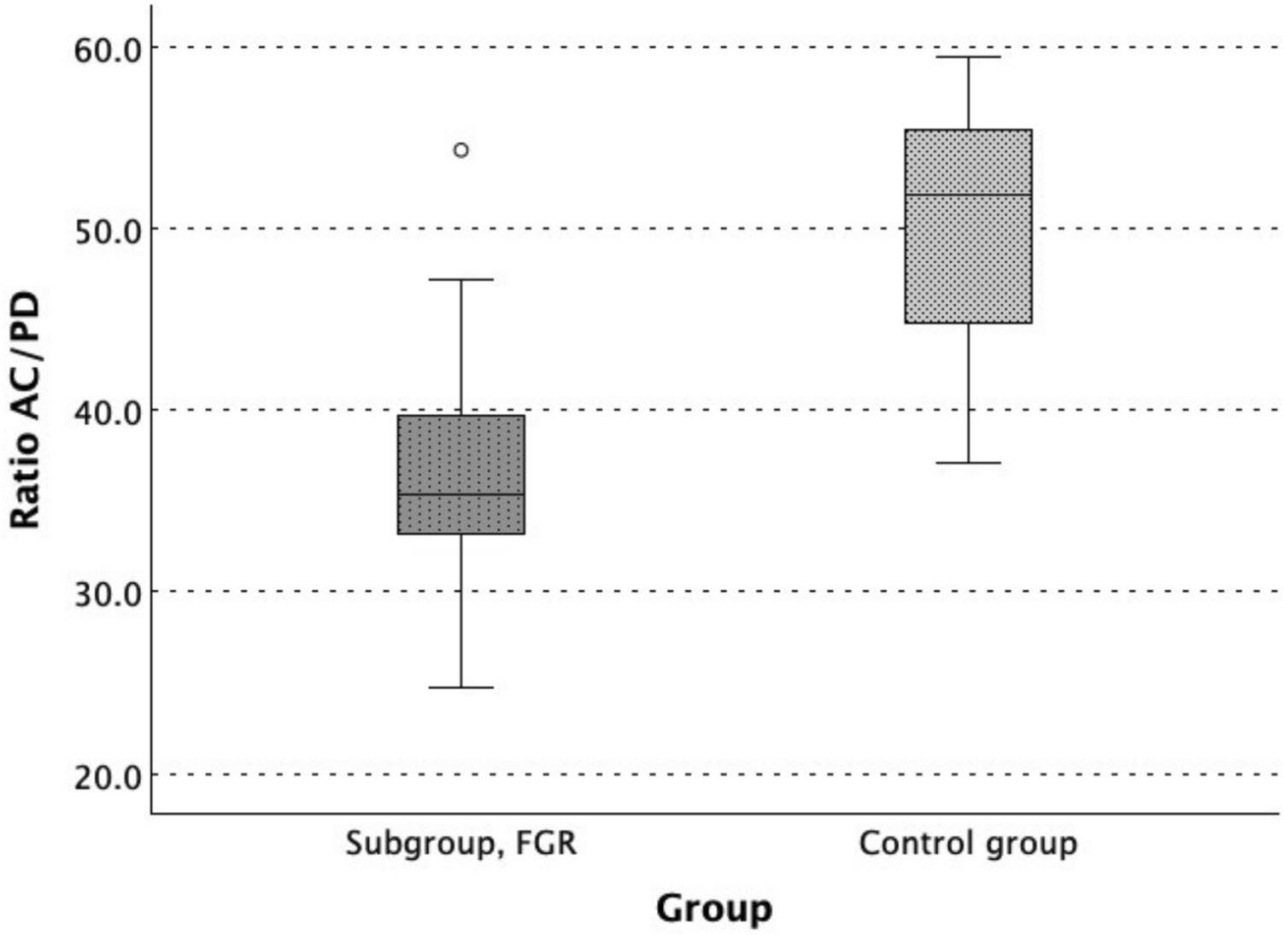
Boxplot of the ratio of abdominal circumference (AC) to pancreas diameter (PD) of subgroup 1, with fetal growth restriction (FGR) (n = 25), in comparison to the corresponding matched control subgroup (n = 25)

AC, the ratio of AC to PD and EFW were smaller in both subgroup 1 (149.2 [14.4] vs. 155.4 [12.6], p 0.002); (35.33 [7.31] vs. 51.88 [11.39], *p* < 0.001); (344.0 [62.5] vs. 382.0 [81.0], p 0.004), and subgroup 2 (150.0 [23.5] vs. 156.3 [19.4], p 0.001); (43.42 [10.71] vs. 50.25 [12.29], *p* < 0.001); (340.0 [83.0] vs. 364.0 [91.3], *p* < 0.001), than in the control groups (Fig. 5).

In multivariable analysis maternal factors such as the BMI, maternal smoking and maternal chronic diseases had no association with the PD (*p* = 0.583, *p* = 0.409, and *p* = 0.943). Low birth weight showed a positive association with the pancreas diameter, with an estimated PD increase of 0.542 mm (95% CI: 0.279–0.806 mm, *p* < 0.001) in case of low birth weight (Table 4).

**Table 4:** Results of the multivariable linear mixed model for pancreatic diameter (PD) in mm to test the influence of covariates

Potential influential factors	Regression coefficient	Lower and upper limit of the 95% confidence interval		*p*-value
Low birthweight, binary (ref: low birthweight = no)	0.542	0.279	0.806	< 0.001
Maternal BMI, categorical kg/m^2^	− 0.005	− 0.024	0.014	0.583
Maternal Smoking, binary (ref: smoking = yes)	− 0.247	− 0.838	0.345	0.409
Maternal chronic disease, binary (ref: chronic disease = yes)	− 0.016	− 0.463	0.431	0.943

*p* values of Wald test

Interrater reliability of PD measurements was good (ICC = 0.758) and intrarater reliability of PD measurements was excellent (ICC = 0.941).

## Discussion

In this study, we found an increased pancreatic diameter between 18 + 0 and 21 + 6 weeks of gestation in low-birth weight fetuses compared to the control group. Furthermore, we introduced the ratio of abdominal circumference to pancreatic diameter as a new sonographic parameter to distinguish between low-birth weight and normal fetuses. The difference in measurements was even more pronounced in fetuses with prenatal signs of FGR. Measurements of the fetal pancreas were feasible with good to excellent intra- and interobserver variabilities.

The fetal pancreas has been shown to be affected in pregnancy complications such as gestational diabetes and FGR. Albeit small in sample size, Gilboa et al. found in their study that an increased circumference of the fetal pancreas at mid second trimester screening ultrasound was predictive of maternal gestational diabetes later in pregnancy [20]. The authors attribute the results to chronic exposure to maternal hyperglycemia, which stimulates fetal β-cell hyperplasia and hypertrophy in response to elevated glucose levels.

The fetal pancreas has also been associated with major genetic abnormalities of the fetus and poor perinatal outcome. In a case series on Beckwith-Wiedemann syndrome, Kagan et al. presented pancreatic hyperechogenicity and hyperplasia as a frequent prenatal feature in affected fetuses, which highlights the importance of the fetal pancreas in high-risk pregnancies [25]. Golbasi et al. examined the circumference of the fetal pancreas in a prospective study of a large cohort of normal pregnancies [23]. While their results on the size of the fetal pancreas in FGR were not statistically significant, they found an enlarged fetal pancreas to be associated with an increased risk of composite adverse neonatal outcomes.

When it comes to FGR, limited nutrient supply to the fetus reduces endocrine tissue and the number of B-cells as well as insulin levels in the animal model. After birth, these newborns display normalized circulating insulin levels with impaired glucose intolerance [26]. When FGR is followed by an early catch-up growth in the animal model, both pancreatic protein expression and morphology are altered and concomitant with glucose intolerance as well as dyslipidemia [27]. With its microvascular structure similar to the adult pancreas, the response of the fetal pancreas to the chronic stress of FGR may include increased capillary permeability an edema as has been described in adult animals exposed to acute or chronic pancreatitis [28, 29].

In their prospective observational study, Arica et al. presented nomograms for the fetal pancreatic circumference and analyzed subgroups of fetuses exposed to either gestational diabetes or FGR [30]. In contrast to previous studies, they could not ascertain a difference in pancreas circumference in gestational diabetes. In FGR, the pancreas circumference was below the 5th centile in the nomograms, but the subgroup contained only 17 fetuses.

The most recent study on the role of the fetal pancreas in FGR was published by Miremberg et al. [31]. In their retrospective study of 69 fetuses with an EFW below the 10th centile, they found a decreased pancreatic circumference in FGR fetuses compared to published nomograms. The Z-score of the pancreatic circumference was − 0.81 ± 1.07 and changes were visible as early as 24 weeks of gestation and persisted through the late pregnancy.

Our study is the largest on the diameter of the fetal pancreas in low birth weight and FGR fetuses so far. However, one of the limitations of our study is its retrospective nature with the possible bias inherent in this study design. While the difference in pancreas diameter was noticeable between the LBW and the control group, the number of fetuses with FGR at time of examination was too small to allow a robust subgroup analysis. Further prospective studies are needed to validate our results.

In our study, we introduce a new method of measuring the fetal pancreas. To our knowledge, a diameter measurement according to the criteria defined above has not been used before and most previous studies have focused on the pancreatic circumference. As we know from studies on the fetal thymus, measuring an organ’s circumference is less reliable and more difficult to reproduce than the measurement of a diameter [32]. Since the pancreas is a similar three-dimensional structure, the measurement of its diameter can be considered more accurate. With its high intra- and interobserver reliability, our method can be considered a solid approach to measuring the fetal pancreas. As we used a standard 2D plane, it can be easily integrated into routine ultrasound.

In conclusion, the diameter of the fetal pancreas, as measured in this study, may be a valuable parameter for the detection of SGA and FGR fetuses. Further prospective studies are warranted to establish cut-off values and consequences for clinical management.

## Data Availability

No datasets were generated or analysed during the current study.
